# Retrieval of a separated instrument using Masserann technique

**DOI:** 10.4103/0972-0707.43417

**Published:** 2008

**Authors:** Arun Kulandaivelu Thirumalai, Mahalaxmi Sekar, Sumitha Mylswamy

**Affiliations:** Department of Conservative Dentistry and Endodontics, SRM Dental College, Chennai, India

**Keywords:** Instrument retrieval, instrument separation, Masserann technique

## Abstract

The fracture of endodontic instruments is a procedural problem creating a major obstacle to normal routine therapy. The separated instrument, particularly a broken file, leads to metallic obstruction in the root canal and impedes efficient cleaning and shaping. When an attempt to bypass such a fragment becomes difficult, it should be retrieved by mechanical devices. Masserann kit is one such device for orthograde removal of intracanal metallic obstructions. These clinical cases demonstrate the usage of Masserann technique in removal of separated instruments in anterior and also the posterior teeth.

The separation of instruments during endodontic therapy is a troublesome incident, and its incidence ranges from 2% to 6% of the cases investigated.[1] Occasionally during nonsurgical root canal therapy, a separated instrument in a canal system may block access to the apical terminus. This instrument is usually some type of file or reamer but can include Gates-Glidden or Peeso drills; lentulo spiral paste fillers; thermomechanical gutta-percha compactors; or the tips of hand instruments, such as explorers or gutta-percha spreaders.[2]

The most common causes for file separation are improper use, limitations in physical properties, inadequate access, root canal anatomy, and possibly manufacturing defects.[2] The separated fragment blocks the access to thorough root canal cleaning and shaping procedure apical to the level of separation or irritates the periapex when it juts out of the root apex. This is significant in a tooth, as it affects the final outcome of the endodontic therapy.[3] Hence an attempt to bypass or retrieve the instrument should be made before leaving it and obturating to the level of separation or embarking upon surgery.

Masserann technique is one among many methods of removal of foreign objects from the root canal.[4] This technique is useful in retrieving broken files, silver points and posts from the root canal; and in general, a success rate of 55% has been reported[5] with the use of this technique.

The armamentarium used consists of long, crown-cutting diamonds (Shofu Preparation Kit, Japan); Gates-Glidden drills (Mani Inc., Japan); slow-speed, contra-angle hand piece (NSK, Japan); and Masserann kit (Micro Mega, France), which contains an assortment of color-coded, end-cutting trephan burs of increasing size which are rotated anticlockwise to create space around the coronal end of the fragment by cutting the surrounding root canal dentin. The extractor is tube like with a plunger rod (stylet) which when screwed inside the extractor locks the exposed coronal end of the fragment against internal embossment just short of the end of the extractor, which can be removed by anticlockwise rotation.

The case reports presented here are about the successful retrieval of a separated file tightly wedged in the root canal dentin of a right lateral incisor, right maxillary first molar, and a left mandibular first molar.

## CASE REPORT

### Case 1

A 28-year-old man was referred to the Department of Conservative Dentistry and Endodontics with acute pain in the right upper back region for the past 3 days.

Radiographic examination revealed inadequate obturation of 16, and there were no periapical changes. During an attempt to remove the gutta-percha, a #25 H-file was separated in the distobuccal canal of 16.

On radiographic examination, it was found that the instrument had separated at the junction of coronal and middle third of the canal [Figure 4a].

Since the efforts of bypassing the fragment went futile, Masserann technique was employed for its retrieval.

**Figure 1:**
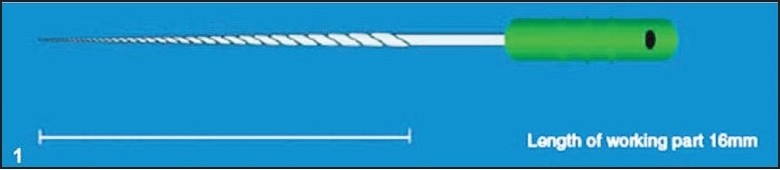
K-file showing the working part of the file (16 mm)

**Figure 2:**
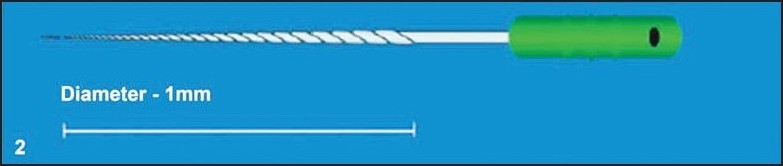
K-file showing the diameter at the fractured end of the file

Radicular access to the coronal end of the fragment was straightened by funneling the root canal with sequential use of Gates-Glidden drills. The remaining part of the separated instrument was examined, and the distance from the tip of the fractured file to D16 (11 mm) was measured and this value was subtracted from the original length, 16 mm, of the file [Figure 1]. This gives the length of the separated fragment remaining in the canal (5 mm). Now the tip diameter at the fractured level was calculated (0.02 + 0.10 = 0.12 mm) [Figure 2].

**Figure 3:**
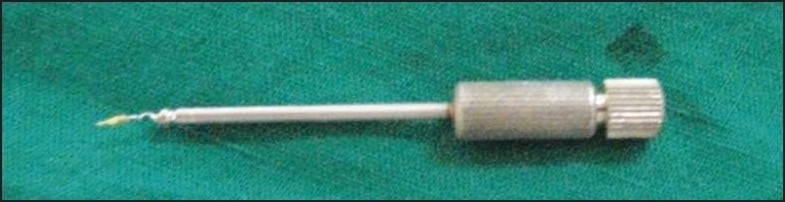
Masserann extractor, along with the removed fragment

**Figure 4:**
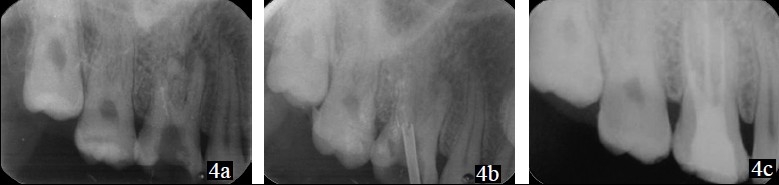
(a) – Radiograph showing the separated file in the distobuccal canal; (b) – Trephan centered over the fragment; (c) – Reobturated 16

The pre-selected trephan with a diameter of 1.2 mm was latched into contra-angle hand piece and run in an anticlockwise direction to create a trough around the coronal end of the fragment by ditching the dentin. The centering of the trephan over the fragment was ensured radiographically. The extractor tube with a diameter of 1.2 mm was slid into the trough to sleeve the fragment [Figure 4b]; and following radiographic confirmation of this, the plunger rod was turned manually inside the extractor tube in a clockwise direction to grip the fragment against its wall. When the tightest grip was felt by the tactile sense, the entire assembly was rotated in an anticlockwise direction to unscrew the fragment from the dentin and withdrawn to see the fragment retrieved [Figure 3].

The canals were enlarged using rotary protaper files, followed by obturation with progutta and zinc oxide eugenol sealer [Figure 4c].

### Case II

A 26-year-old man was referred to the Department of Conservative Dentistry and Endodontics with a dull pain in the left lower back region for the past 1 month.

Radiographic examination revealed dental caries in 36, 37. After elaborate history-taking and thorough clinical examination, it was diagnosed that 36, 37 had dental caries with chronic irreversible pulpitis. Root canal treatment was planned for 36, 37.

**Figure 5:**
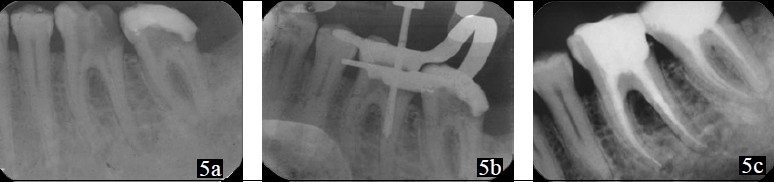
(a) – Radiograph showing the separated file in the distolingual canal; (b) – Extractor with plunger sleeving and gripping the fragment; (c) – Post-obturation 36, 37

Access opening was done under rubber dam in 36, 37. Four canals were located in 36, 37. Working length was determined. During cleaning and shaping, a #25 stainless steel K-file was separated in distolingual canal in 36. A radiograph was taken to confirm the level of separation of the instrument [Figure 5a]. The instrument was found to be separated at the junction of coronal and middle third of the root canal. Masserann technique was used for retrieval of the instrument [Figure 5b]; and after several attempts, the instrument was retrieved successfully.

Cleaning and shaping was completed in 36, 37 with rotary instruments and the canal was obturated with progutta percha and zinc oxide eugenol sealer [Figure 5c].

### Case III

A 35-year-old man was referred for the endodontic treatment of maxillary left lateral incisor to the Department of Conservative Dentistry and Endodontics.

Radiographic examination revealed dental caries in 22. After thorough history-taking and clinical examination with pulp testing, it was diagnosed as dental caries with chronic irreversible pulpitis.

**Figure 6:**
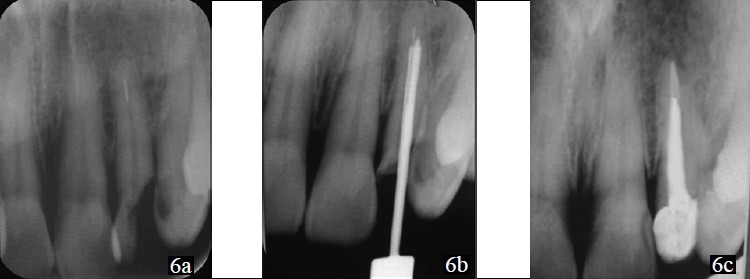
(a) – Radiograph showing the separated file; (b) – Extractor with plunger sleeving and gripping the fragment; (c) – Post-endodontic restoration given

Access opening was done and working length determined. During cleaning and shaping, a #35 NiTi hand file was separated at the junction of middle and apical third of the root canal [Figure 6a]. Masserann kit was used to remove the fractured segment [Figure 6b].

Routine root canal treatment was done in 22, and post endodontic restoration was given [Figure 6c].

## DISCUSSION

Intracanal separation of instruments usually prevents access to the apex, impedes thorough cleaning and shaping of the root canal, and thus may compromise the outcome of endodontic treatment and reduce the chances of successful retreatment.[5,6] In such cases, prognosis following an endodontic therapy depends on the condition of the root canal (vital or nonvital), tooth (symptomatic or asymptomatic, with or without periapical pathology), level of cleaning and shaping at the time of separation, the level of separation in the canal; and is generally lower than that with normal endodontic treatment.[1]

Hence every attempt should be made to bypass or retrieve the separated instrument. The orthograde retrieval depends on cross-sectional diameter, length, curvature of the canal; dentin thickness and morphology of the root; composition, cutting action (clockwise or counterclockwise) of the instrument; length, location, and amount of binding or impaction of the fragment in the canal.[5] Masserann kit has been used for over 30 years as a device for removing broken instruments, and a success rate of 73% and 44% has been reported regarding its use in anterior and posterior teeth respectively.[6] However, it needs frequent radiographic monitoring.[6] It has limited application in teeth with thin roots, curved roots or in retrieving instruments which fractured apically, as the use of relatively large and rigid trephans leads to removal of considerable amount of root dentin and weakening of the teeth or risk of perforation.[3]

However, Masserann kit is very useful in removing metal obstructions from anterior teeth having thick, straight roots. Moreover, the locking mechanism of the extractor provides considerable retention in gripping and dislodging an obstruction which is tightly wedged in the canal. In all of the above-mentioned cases, obtaining a straight-line access to the fragment facilitated centering of the trephan over the fragment. This ensured circumferential freeing of the coronal end of the fragment with safe cutting of the peripheral dentin around the fragment. This in turn promoted tight gripping of the fragment and its retrieval along the long axis of the root, thus allowing regular retreatment.

It has been reported in the literature that it is difficult to use this technique for posterior teeth.[7] But our attempts to remove the separated file in the posterior teeth using Masserann kit proved to be successful. All the cases were done under rubber dam. In case report I, since the wing of the clamp obstructed the appearance of the separated segment in the radiograph, the clamp was removed and the radiograph was taken. In the case report III, as the tooth was grossly destructed, wedgets were used instead of clamps to retain the rubber dam. Prevention of the instrument separation is the best strategy to avoid any stress and anxiety associated with it.[8] In case of separation, safe retrieval or bypassing should be carried out. Among the retrieval methods, Masserann technique is technique sensitive and time consuming[9]; yet by tactful applicability, within its clinical limitations, coupled by the skill of the operator, separated files were retrieved from maxillary lateral incisor, maxillary and mandibular molar. Nevertheless, use of ultrasonics, coupled with the dental operating microscope, makes it more effective in selected cases.[10]
